# Post-discharge Outcomes of COVID-19 Patients in a Tertiary Care Hospital: A Descriptive Cross-sectional Study

**DOI:** 10.31729/jnma.8530

**Published:** 2024-04-30

**Authors:** Naveen Phuyal, Ganesh Bhandari, Lee Budhathoki, Kshitij Kumar Shrestha, Lochana Shrestha, Poonam Singh

**Affiliations:** 1 Department of Community Medicine, Nepalese Army Institute of Health Sciences, College of Medicine, Sanobharyang, Kathmandu, Nepal; 2 School of Health and Allied Sciences, Pokhara University, Nepal; 3 Department of Anatomy, Nepalese Army Institute of Health Sciences, College of Medicine, Sanobharyang, Kathmandu, Nepal

**Keywords:** *COVID-19*, *mental health*, *Nepal*, *patient outcome assessment*

## Abstract

**Introduction:**

Understanding the post-discharge outcomes of COVID-19 patients is essential for informed healthcare planning and support services. This study aimed to assess the physical health status of COVID-19 patients three months after discharge from a tertiary care hospital in Kathmandu, Nepal.

**Methods:**

A descriptive follow-up study design was used, involving 200 COVID-19 discharged patients. Data were collected from healthcare facilities and participants through structured questionnaires and telephonic interviews. The study duration was between November 2020 to April 2021.

**Results:**

Persistence of COVID-19-related symptoms was reported by 49 (24.50%) of participants reported at follow-up, while 41 (20.50%) indicated previous symptoms from discharge.

**Conclusions:**

After discharge, most of patient returned to normal activities within three months. Persistence of symptoms and test positive rate was less in those patients.

## INTRODUCTION

The global COVID-19 pandemic has presented an exceptional challenge to healthcare systems worldwide.^1^ In response to this crisis, numerous studies have aimed to understand various aspects of the disease, from its clinical manifestations to therapeutic interventions.^2-4^ However, the focus on the post-discharge phase remains limited.

As COVID-19 affected millions of individuals globally, the management and care of discharged patients have gained increasing importance. While much attention has been directed toward acute disease management, there is a critical need to examine the longer-term consequences, recovery trajectories, and associated challenges patients face after leaving the hospital.^5^ This study provides a comprehensive analysis of post-discharge outcomes of COVID-19-recovered patients.

The aim of this study was to assess the post-discharge outcomes of COVID-19 patients, in terms of their physical health in a tertiary care hospital and one of the isolation centers in Kathmandu.

## METHODS

In this descriptive follow-up study, we examined the post-discharge outcomes of COVID-19 patients in the context of Kathmandu Valley, Nepal. Data were collected from a tertiary healthcare facility: the COVID-19 facility at Shree Birendra Hospital (SBH) and one of the isolation centers operated by the Nepal Army. Non-probability purposive sampling was done to select the study site. A census was done for the participants who met specific inclusion criteria over a six-month duration between November 2020 to April 2021. To be eligible, participants had to be COVID-19 patients admitted and discharged from either the COVID-19 facility at SBH or the designated isolation center of SBH to their homes. Additionally, they were required to have been discharged within three months before the date of research approval and must have been permanent residents of Kathmandu. Ethical approval was obtained from the Institutional Review Committee (Reference number: 245), and informed consent was secured from all participants. Data collection involved a retrospective record review, structured questionnaires administered to participants, and prospective telephonic interviews. Outcome of physical health for this study is taken as persistence of symptoms related to illness and persistence of previous symptoms which was defined as symptoms which were present before and still present on follow up while return to normal activity is taken as getting back to regular daily work.

Descriptive analysis using IBM SPSS (Statistical Package for the Social Sciences) was conducted to comprehensively assess the post-discharge outcomes, shedding light on the physical, psychological, and social dimensions of COVID-19 recovery in the Kathmandu Valley.

## RESULTS

There were 200 patients enrolled in the study out of which 133 (66.50%) were male. There were 156 (78%) patient treated in isolation ward centres, the symptoms on admission included fever 106 (53%) and dry cough 92 (46%). Comorbidities like hypertension 53 (26.50%) and diabetes 30 (15%) were prevalent among participants (Table 1). There were 45 (22.50%) patient who got symptomatic again after discharge and came into contact for medical service, out of those 39 (86.67%) came back to hospital, among those 10 (22.22%) patients got admitted, 17 (37.70%) patient came into contact within 15 days of discharge (Table 2).

**Table 1:** Demographic and Clinical Characteristics of COVID-19 Patients (n= 200).

Variables		n (%)
Age	Less than 25	54 (27)
	25-35	51 (25.50)
	36-57	50 (25)
	more than 57	45 (22.50)
	Median age=38.25 years, Interquartile range=32 years	
Sex	Female	67 (33.50)
	Male	133 (66.50)
Place of treatment^*^	Isolation ward centre	156 (78)
	Treated with supplemented oxygen	66 (33)
	Treated in ICU	40 (20)
	Treated with invasive mechanical ventilation	4 (2)
Symptoms on admission^*^	Fever	106 (53)
	Dry cough	92 (46)
	Dyspnoea	55 (27.50)
	Myalgia	37 (18.50)
	Headache	27 (13.50)
	Sore throat	13 (6.50)
	Diarrhea	14 (7)
	Fatigue	22 (11)
	Loss of taste	22 (11)
	Loss of odour	16 (8)
	Anorexia	14 (7)
	Vomiting	6 (3)
	None of any	14 (7)
Comorbidities present^*^	Hypertension	53 (26.50)
	Diabetes	30 (15)
	Chronic kidney disease	11 (5.50)
	Chronic obstructive pulmonary disease	12 (6)
	Chronic vascular disease	10 (5)
	Chronic liver disease	2 (1)

* *Multiple responses*

**Table 2:** Means and duration of follow up after discharge (n= 45).

Variables		n (%)
	Hospital	39 (86.67)
	Clinic	1 (2.22)
Place/means of follow up done	Telephone	3 (6.67)
	Video conferencing	1 (2.22)
	Home Health services	1 (2.22)
Duration of follow-up care after discharge	< 15 days	17 (37.70)
	15-30 Days	16 (35.50)
	> 30 Days	12 (26.67)

In 151 (75.50%) there was no persistence of COVID-19 symptoms, 179 (89.50%) did not test positive again 198 (99%) (Table 3).

**Table 3:** Post-Discharge Outcomes and Well-being of COVID-19 Patients (n= 200).

Outcomes		n (%)
Persistence of symptoms	Yes	49 (24.50)
related to illness	No	151 (75.50)
Persistence of previous	Yes	41 (20.50)
symptoms	No	159 (79.50)
Returned to normal activity	Yes	179 (89.50)
	No	21 (10.50)
Tested positive again	Yes	2 (1)
	No	198 (99)

## DISCUSSION

This study assessed the post-discharge outcomes of COVID-19 patients. Our findings shed light on the health status of individuals after being discharged from COVID-19 treatment facilities. A large proportion of participants did not report the persistence of COVID-19-related symptoms (75.5%) or previous symptoms (79.5%) three months after discharge. This observation aligns with some previous studies indicating that most COVID-19 patients experience a gradual improvement in their physical health after the acute phase of the illness.^6,7^ These findings are supportive, suggesting that many patients recover well in terms of physical health within a few months.

No physical disabilities were found among the participants after the three-month follow-up period. No participant reported social health issues which suggest that, in this group, COVID-19 did not lead to long-term physical disabilities or significant social disruptions. Our study population being affiliated to the army could be the reason for this. However, it is crucial to acknowledge that the social and economic consequences of the pandemic may vary widely across populations and regions, and longer-term studies may be needed to understand the extent of these impacts fully.^10^

Post-discharge outcome could be dependent on the severity of various comorbidities like Hypertension, Diabetes mellitus, Chronic kidney disease, Chronic Obstructive Pulmonary Disease and Chronic liver disease, the outcome being dependent on the severity of these comorbidities, which was not studied in this study which is a limitation of this study. The study included a larger population of serving soldiers, potentially introducing health worker bias. Response bias may occur as participants might underreport symptoms due to concerns about their medical category. The study on patients who survived COVID-19 could be responsible for survivorship bias. Data was collected from a hospital and its isolation center so the clinical profile can be generalized to such a setting, however outcome needs to be further analysed by outcome based sample size calculation and comparing it with a comparative arm.

## CONCLUSIONS

Most of the patient has returned to normal activities within three months of discharge. Persistence of symptoms and test positive rate was less in those patients.
